# Machine learning and bioinformatics to identify 8 autophagy-related biomarkers and construct gene regulatory networks in dilated cardiomyopathy

**DOI:** 10.1038/s41598-022-19027-5

**Published:** 2022-09-02

**Authors:** Fengjun Zhang, Mingyue Xia, Jiarong Jiang, Shuai Wang, Qiong Zhao, Cheng Yu, Jinzhen Yu, Dexian Xian, Xiao Li, Lin Zhang, Yuan Liu, Min Peng

**Affiliations:** 1 College of Acupuncture and Massage, Shandong University of Traditional Chinese Medicine, Jinan, China; 2 College of Traditional Chinese Medicine, Shandong University of Traditional Chinese Medicine, Jinan, China; 3 Department of Cardiology, PLA Rocket Force Characteristic Medical Center, Beijing, China; 4 Department of Pediatric Surgery, Shandong Provincial Hospital affiliated to Shandong First Medical University, Jinan, China; 5 Department of Traditional Chinese Medicine, Shandong Provincial Hospital affiliated to Shandong First Medical University, Jinan, China; 6 Department of Traditional Chinese Medicine Classics, Shandong University of Traditional Chinese Medicine Affiliated Hospital, Jinan, Shandong China; 7 First Clinical Medical College, Shandong University of Traditional Chinese Medicine, Jinan, China; 8 Department of Cardiology, Shandong University of Traditional Chinese Medicine Affiliated Hospital, Jinan, Shandong China; 9 Department of Clinical Pharmacy, Shaoxing People’s Hospital, Shaoxing Hospital, Zhejiang University School of Medicine, Shaoxing, China

**Keywords:** Bioinformatics, Cardiac hypertrophy

## Abstract

Dilated cardiomyopathy (DCM) is a condition of impaired ventricular remodeling and systolic diastole that is often complicated by arrhythmias and heart failure with a poor prognosis. This study attempted to identify autophagy-related genes (ARGs) with diagnostic biomarkers of DCM using machine learning and bioinformatics approaches. Differential analysis of whole gene microarray data of DCM from the Gene Expression Omnibus (GEO) database was performed using the NetworkAnalyst 3.0 platform. Differentially expressed genes (DEGs) matching (|log2FoldChange ≥ 0.8, p value < 0.05|) were obtained in the GSE4172 dataset by merging ARGs from the autophagy gene libraries, HADb and HAMdb, to obtain autophagy-related differentially expressed genes (AR-DEGs) in DCM. The correlation analysis of AR-DEGs and their visualization were performed using R language. Gene Ontology (GO) enrichment analysis and combined multi-database pathway analysis were served by the Enrichr online enrichment analysis platform. We used machine learning to screen the diagnostic biomarkers of DCM. The transcription factors gene regulatory network was constructed by the JASPAR database of the NetworkAnalyst 3.0 platform. We also used the drug Signatures database (DSigDB) drug database of the Enrichr platform to screen the gene target drugs for DCM. Finally, we used the DisGeNET database to analyze the comorbidities associated with DCM. In the present study, we identified 23 AR-DEGs of DCM. Eight (*PLEKHF1*, *HSPG2*, *HSF1*, *TRIM65*, *DICER1*, *VDAC1*, *BAD*, *TFEB*) molecular markers of DCM were obtained by two machine learning algorithms. Transcription factors gene regulatory network was established. Finally, 10 gene-targeted drugs and complications for DCM were identified.

## Introduction

Dilated cardiomyopathy (DCM), which manifests clinically as ventricular dilatation and impaired progressive systolic diastole, is one of the most prevalent disease worldwide. It has a heterogeneous etiology, with viral infections, inflammatory reactions, genetic factors^1^, etc. It can also cause arrhythmias and atrioventricular block, resulting in sudden cardiac death and heart failure. These circumstances often occur with a poor prognosis^2^. It is reported that men with DCM have a higher mortality rate than women patients^3^. Endomyocardial biopsy (EMB) is the gold standard for the diagnosis of myocarditis and DCM. However, in clinical practice, DCM is not diagnosed and treated promptly, considering the high cardiac complications of performing EMB and treatment limitation^4^. Therefore, developing innovative, non-invasive biomarkers for DCM is essential to improve diagnostic accuracy.

Autophagy is a cellular self-degradation process that removes errant proteins and damaged organelles. It also eliminates intracellular pathogens and is often considered a survival mechanism^5^. Numerous studies have shown that autophagy genes are involved in various phenotypes and human diseases^6^, including neurodegenerative diseases ^7^, liver diseases^8^, muscle diseases^9^, cancer ^10^, and cardiac diseases^11^. Evidence shows that autophagy is essential in maintaining cardiomyocyte homeostasis^12^ and regulating the prognostic efficacy of cardiac diseases. In addition, an increasing number of animal models and clinical studies have reported the involvement of autophagy-related genes (ARGs) in the ventricular remodeling process, which is related to the mechanism of action of DCM^13,14^. However, ARGs' diagnostic performance and prognostic efficacy in DCM have not been fully elucidated.

In this study, we downloaded gene expression profile data of DCM from the Gene Expression Omnibus (GEO) database, applied bioinformatics to search for AR-DEGs in DCM, and visualized the correlation between genes. Subsequently, gene enrichment analysis was performed on tagging gene functions and exploring pathogenesis. Machine learning algorithms were afterward executed to filter and identify diagnostic biomarkers of DCM. In addition, based on the diagnostic biomarkers of DCM, transcription factors gene regulatory network and gene-targeted drugs were predicted to provide ideas for clinical precision therapy and experimental studies. The DisGeNET database was used for association analysis of DCM with other related diseases to provide a transcriptomic basis for further investigation of the potential pathogenesis of the disease.

**Figure 1:**
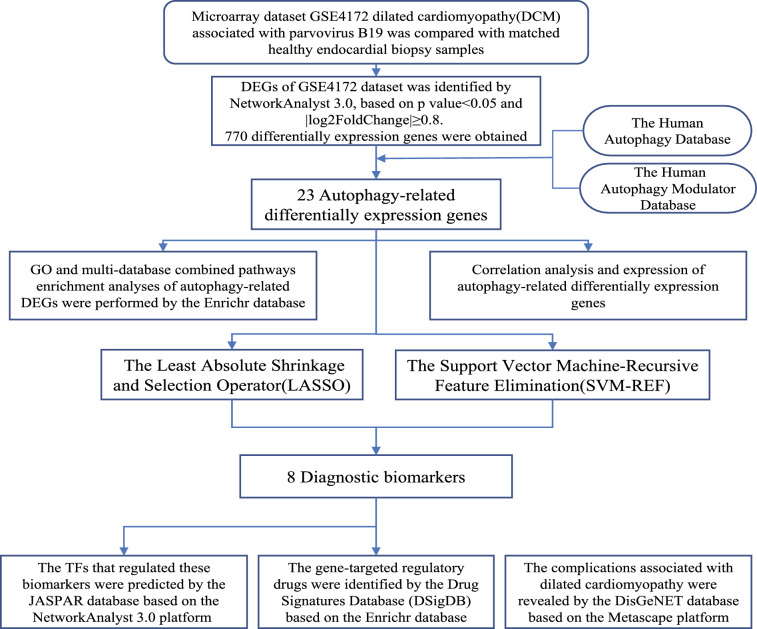
Workflow diagram of the current study. GO, go ontology; TFs, transcription factors.

The flow chart of this study was shown in Fig. 1.

## Results

### Identification of autophagy-related differentially expressed genes (AR-DEGs) for dilated cardiomyopathy (DCM)

**Figure 2:**
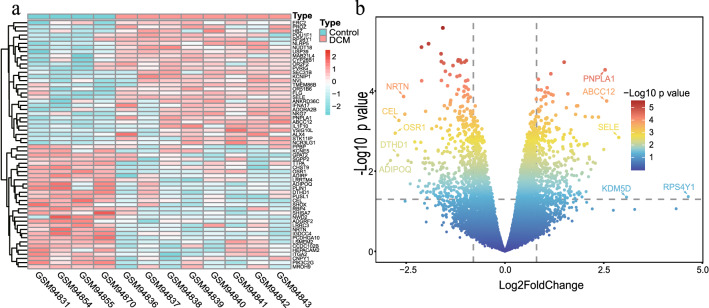
DEGs differential analysis of GSE4172 dataset. (**a**) Heatmap of DEGs in GSE4172 dataset (n = 60, p < 0.05, |log2 FoldChange|≥ 0.8). (**b**) Asymptotic volcano map of gene expression in the GSE4172 dataset. The two vertical lines indicated gene expression ploidy changes > 0.8 and < -0.8, respectively, and the horizontal line indicated a p value of 0.05. The color of the dots represented the level of the p value. The top 10 significantly expressed genes among the DEGs were labeled on the graph.

The GSE4172 dataset was used to screen for DEGs in DCM. Based on the threshold set to |log2FoldChange|≥ 0.8, p-value < 0.05, 770 DEGs were acquired, containing 366 up-regulated genes and 404 down-regulated genes. In addition, the heatmap (Fig. 2a) showed the expression of the top 60 DEGs and the asymptotic volcano plot (Fig. 2b) showed the distribution of DEGs.

**Figure 3:**
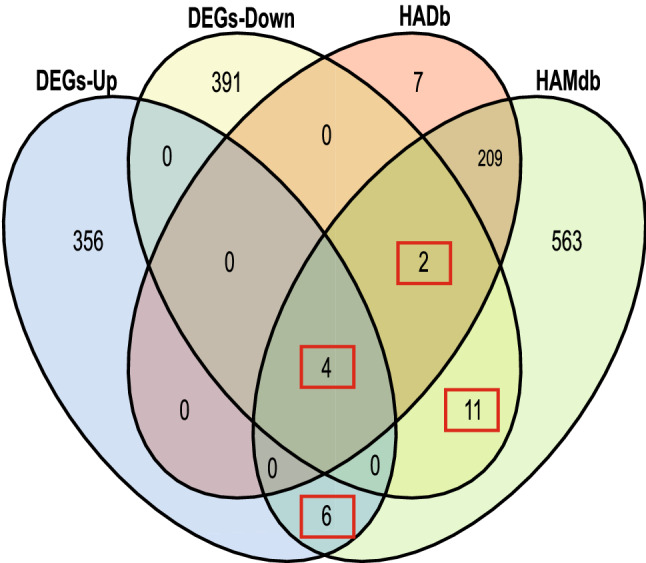
AR-DEGs were shown by Venn diagram. 366 DEGs-Up and 404 DEGs-Down were intersected with 232 and 796 autophagy-associated genes from the HADb and HAMDb autophagy gene pools, with 23 genes being identical. The number of intersecting genes was marked in the red box. DEGs-Up, differentially expressed up-regulated genes; DEGs-Down, differentially expressed down-regulated genes.

803 ARGs were obtained through two autophagy-related gene databases, HADb and HAMdb. The Venn diagram obtained by the Omicshare online tool demonstrated 23 AR-DEGs of DCM (*ADIPOQ*, *TRIM17*, *PPFIA4*, *CAPN12*, *PLEKHF1*, *RCAN1*, *RAB12*, *CXCR4*, *HSPG2*, *EIF4EBP1*, *HSF1*, *ZC3H12A*, *PRKAB1*, *TRIM65*, *ARSA*, *GABARAPL1*, *DICER1*, *VDAC1*, *CHMP4B*, *AGTR1*, *BAD*, *TFEB*, *AP2M1*) (Fig. 3). The relevant functions of 23 AR-DEGs were shown in Supplementary Table S1.

**Figure 4:**
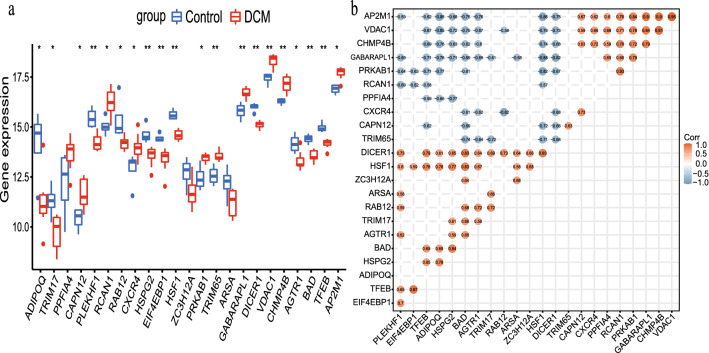
23 AR-DEGs in dilated cardiomyopathy (DCM) group and control group and their correlation. (**a**) Box plot of the expression levels of 23 DEGs-Down in the DCM and control groups. The blue box plots above the corresponding gene names indicated expression in control groups, whereas the red box plots indicated expression in DCM groups. (**b**) Correlation heatmap of 23 AR-DEGs. The color within the circle shape and the magnitude of the correlation value represented the strength of the correlation; red represented positive correlation and blue represented negative correlation. The darker the color, the larger the absolute value of the correlation value represented a stronger correlation.

Correlation matrix analysis of 23 AR-DEGs and the expression of these genes in the disease and control groups were demonstrated in Fig. 4a. The absolute values of relative coefficients between genes exceeding 0.5 were considered to be of typical significance and were labeled in Fig. 4b. Moreover, some genes showed a strong association with others.

### Gene Ontology (GO), Pathway Enrichment Analysis

GO analysis and multiple databases (KEGG, Wikipathway, Bioplanet, Reactome) pathway analysis were implemented through the Enrichr database. Three categories of GO analysis were obtained by clustering AR-DEGs of DCM, namely biological process (BP), chromosomal location (CC), and molecular function (MF). The top ten terms of each category were predicted in Table 1.

**Table 1:** GO category, GO pathways, corresponding p-values, and AR-DEGs.

Go category	GO pathways	GO ID	p-value	AR-DEGs
Biological process	Regulation of autophagy	GO:0010506)	5.24E−09	*PLEKHF1*;*BAD*;*ZC3H12A*;*TFEB*;*VDAC1*; *PRKAB1*;*TRIM65*
	Positive regulation of autophagy	GO:0010508)	5.2E−08	*PLEKHF1*;*BAD*;*ZC3H12A*;*TFEB*;*TRIM65*
	Positive regulation of cellular catabolic process	GO:0031331)	4.93E−07	*PLEKHF1*;*BAD*;*ZC3H12A*;*TFEB*;*TRIM65*
	Positive regulation of cold-induced thermogenesis	GO:0120162)	4.29E−06	*ADIPOQ*;*HSF1*;*CXCR4*;*PRKAB1*
	Positive regulation of metabolic process	GO:0009893)	7.87E−06	*ADIPOQ*;*HSF1*;*CXCR4*;*PRKAB1*
	Macroautophagy	GO:0016236)	9.99E−06	*GABARAPL1*;*CHMP4B*;*VDAC1*;*PRKAB1*
	Response to sodium arsenite	GO:1903935)	1.26E−05	*HSF1*;*ZC3H12A*
	Cellular response to sodium arsenite	GO:1903936)	1.26E−05	*HSF1*;*ZC3H12A*
	Cellular response to salt	GO:1902075)	1.89E−05	*HSF1*;*ZC3H12A*
	Negative regulation of tumor necrosis factor production	GO:0032720)	1.95E−05	*ADIPOQ*;*ZC3H12A*;*DICER1c*
Molecular function	Low-density lipoprotein particle receptor binding	GO:0050750)	0.000315	*HSPG2*;*AP2M1*
	Lipoprotein particle receptor binding	GO:0070325)	0.00047	*HSPG2*;*AP2M1*
	Endoribonuclease activity	GO:0004521)	0.000694	*ZC3H12A*;*DICER1*
	Regulatory RNA binding	GO:0061980)	0.000961	*ZC3H12A*;*DICER1*
	Endonuclease activity	GO:0004519)	0.001559	*ZC3H12A*;*DICER1*
	Kinase binding	GO:0019900)	0.001742	*BAD*;*HSF1*;*VDAC1*;*PRKAB1*
	Protein kinase binding	GO:0019901)	0.002446	*BAD*;*HSF1*;*VDAC1*;*PRKAB1*
	Ribonuclease activity	GO:0004540)	0.002595	*ZC3H12A*;*DICER1*
	C-X-C chemokine receptor activity	GO:0016494)	0.005737	*CXCR4*
	Intronic transcription regulatory region sequence-specific DNA binding	GO:0001161)	0.005737	*HSF1*
Cellular component	lysosome	GO:0005764)	7.4E−07	*ARSA*;*PLEKHF1*;*RAB12*;*TFEB*;*CXCR4*; *HSPG2*;*AP2M1*
	Lytic vacuole	GO:0000323)	0.000105	*ARSA*;*PLEKHF1*;*RAB12*;*CXCR4*
	Lytic vacuole membrane	GO:0098852)	0.003419	*PLEKHF1*;*TFEB*;*AP2M1*
	Lysosomal lumen	GO:0043202)	0.00436	*ARSA*;*HSPG2*
	Endosome membrane	GO:0010008)	0.005917	*PLEKHF1*;*RAB12*;*AP2M1*
	Lysosomal membrane	GO:0005765)	0.006172	*PLEKHF1*;*TFEB*;*AP2M1*
	Nuclear stress granule	GO:0097165)	0.006881	*HSF1*
	AP-2 adaptor complex	GO:0030122)	0.008023	*AP2M1*
	Clathrin coat of endocytic vesicle	GO:0030128)	0.008023	*AP2M1*
	Mitochondrial outer membrane	GO:0005741)	0.009136	*BAD*;*VDAC1*

Based on the number of gene interactions, BP was mainly focused on the regulation of autophagy, positive regulation of autophagy, positive regulation of the cellular catabolic process, and macroautophagy. For cellular components, lysosome and lytic vacuole were significantly associated with autophagy-related differential genes, ultimately pointing to inflammatory cardiomyopathy in response to the human heart. Molecular functional studies revealed that AR-DEGs were most concentrated in low-density lipoprotein particle receptor binding. A similar concentration level could be found in lipoprotein particle receptor blinding and endoribonuclease activity.

Notably, the results of the pathway analysis in this study were joint (Table 2). Through the previously set database, the Longevity regulating pathway, Macroautophagy, PI3K-AKT-mTOR signaling pathway, therapeutic opportunities, and AMPK signaling were identified as the top pathways.

**Table 2:** Top 10 pathways from KEGG, BioPlanet, Reactome, WikiPathways databases and their corresponding p-values and genes for AR-DEGs.

Databases	Pathways	p-value	Genes
KEGG	Longevity regulating pathway	2.12E−04	*ADIPOQ*;*EIF4EBP1*;*PRKAB1*
	AMPK signaling pathway	3.42E−04	*ADIPOQ*;*EIF4EBP1*;*PRKAB1*
	Apelin signaling pathway	5.04E−04	*GABARAPL1*;*AGTR1*;*PRKAB1*
	Insulin signaling pathway	5.04E−04	*BAD*;*EIF4EBP1*;*PRKAB1*
	cGMP-PKG signaling pathway	8.96E−04	*BAD*;*AGTR1*;*VDAC1*
	Calcium signaling pathway	0.002531	*AGTR1*;*CXCR4*;*VDAC1*
	Acute myeloid leukemia	0.002673	*BAD*;*EIF4EBP1*
	Mitophagy	0.002752	*GABARAPL1*;*TFEB*
	Adipocytokine signaling pathway	0.002832	*ADIPOQ*;*PRKAB1*
	Endocytosis	0.002905	*CXCR4*;*CHMP4B*;*AP2M1*
BioPlanet	AMPK signaling	6.34E−05	*ADIPOQ*;*EIF4EBP1*;*PRKAB1*
	Mitochondrial pathway of apoptosis: BH3-only Bcl-2 family	1.83E−04	*CAPN12*;*BAD*;*VDAC1*
	Phosphoinositides and their downstream targets	3.73E−04	*BAD*;*AP2M1*
	PKB-mediated events	4.70E−04	*EIF4EBP1*;*PRKAB1*
	TOR signaling	6.94E−04	*EIF4EBP1*;*PRKAB1*
	Endocytosis	0.001527	*CXCR4*;*CHMP4B*;*AP2M1*
	Calcineurin-dependent NFAT signaling role in lymphocytes	0.00181	*RCAN1*;*BAD*
	Acute myeloid leukemia	0.001943	*BAD*;*EIF4EBP1*
	ERK1/ERK2 MAPK pathway	0.002518	*BAD*;*EIF4EBP1*
	Adipocytokine signaling pathway	0.002673	*ADIPOQ*;*PRKAB1*
Reactome	Macroautophagy Homo sapiens R-HSA-1632852	6.07E−05	*GABARAPL1*;*CHMP4B*;*PRKAB1*
	Cellular responses to stress Homo sapiens R-HSA-2262752	7.49E−04	*GABARAPL1*;*HSF1*;*CHMP4B*;*PRKAB1*
	mTOR signalling Homo sapiens R-HSA-165159	9.13E−04	*EIF4EBP1*;*PRKAB1*
	PKB-mediated events Homo sapiens R-HSA-109703	9.61E−04	*EIF4EBP1*;*PRKAB1*
	Disease Homo sapiens R-HSA-1643685	0.001206	*BAD*;*CXCR4*;*CHMP4B*;*HSPG2*;*AP2M1*
	HIV Infection Homo sapiens R-HSA-162906	0.002028	*CXCR4*;*CHMP4B*;*AP2M1*
	PI3K Cascade Homo sapiens R-HSA-109704	0.003693	*EIF4EBP1*;*PRKAB1*
	Degradation of the extracellular matrix Homo sapiens R-HSA-1474228	0.006547	*CAPN12*;*HSPG2*
	Infectious disease Homo sapiens R-HSA-5663205	0.007144	*CXCR4*;*CHMP4B*;*AP2M1*
	Nef Mediated CD8 Down-regulation Homo sapiens R-HSA-182218	0.008023	*AP2M1*
WikiPathway	PI3K-AKT-mTOR signaling pathway and therapeutic opportunities WP3844	5.28E−06	*BAD*;*TFEB*;*EIF4EBP1*
	Leptin and adiponectin WP3934	5.66E−05	*ADIPOQ*;*PRKAB1*
	AMP-activated protein kinase (AMPK) signaling WP1403	6.62E−05	*ADIPOQ*;*EIF4EBP1*;*PRKAB1*
	The influence of laminopathies on Wnt signaling WP4844	7.36E−04	*ADIPOQ*;*DICER1*
	Target Of Rapamycin (TOR) Signaling WP1471	7.78E−04	*EIF4EBP1*;*PRKAB1*
	Synaptic signaling pathways associated with autism spectrum disorder WP4539	0.001499	*EIF4EBP1*;*PRKAB1*
	RAC1/PAK1/p38/MMP2 Pathway WP3303	0.002752	*BAD*;*EIF4EBP1*
	Peptide GPCRs WP24	0.003249	*AGTR1*;*CXCR4*
	Leptin signaling pathway WP2034	0.003424	*BAD*;*EIF4EBP1*
	IL-18 signaling pathway WP4754	0.003602	*BAD*;*ADIPOQ*;*ZC3H12A*

**Figure 5:**
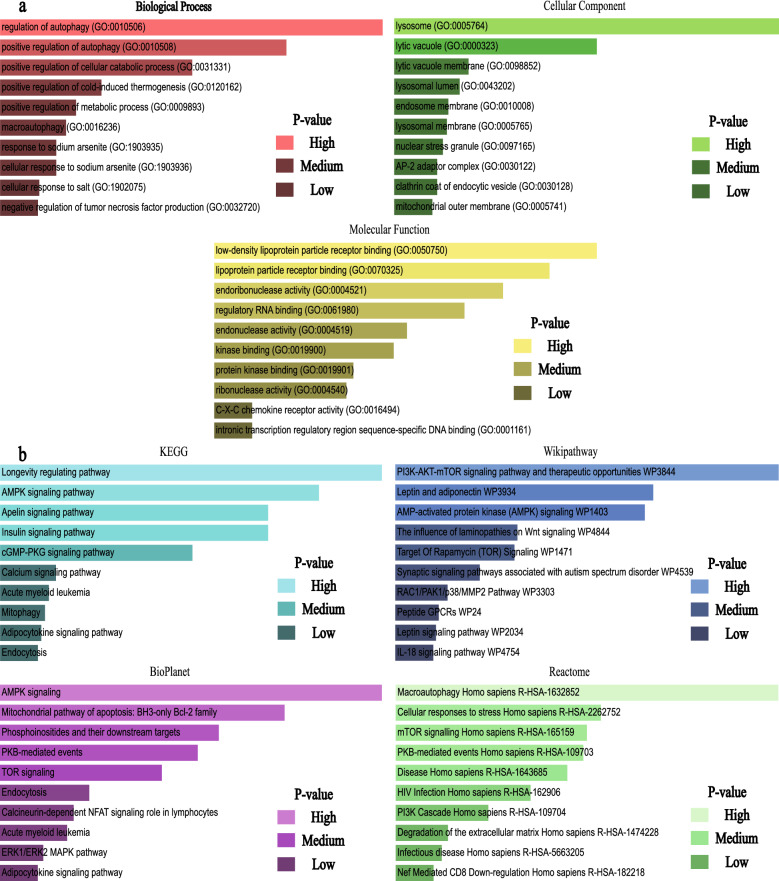
(**a**) Identification results of GO terms related to biological processes, cellular components and molecular functions based on gene enrichment analysis. Higher p value indicated a higher number of genes involved in this GO ontology. (**b**) Identification of results from combined multi-pathway analysis by KEGG, WikiPathways, BioPlanet and Reactome.

A comparison of GO terms was presented in Fig. 5a. Figure 5b provided pathway analysis from multiple databases.

### Machine learning screened for autophagy-related biomarkers of DCM

**Figure 6:**
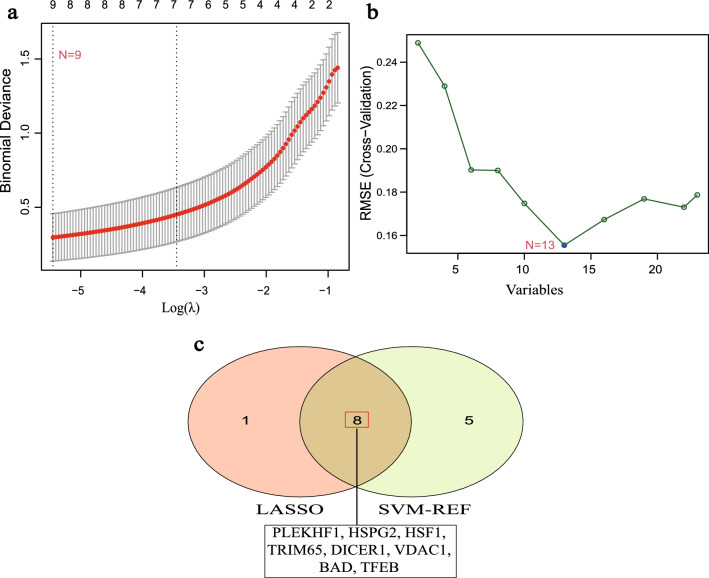
Screening of diagnostic biomarkers for DCM by machine learning algorithms. (**a**) Screening of optimal genes by LASSO regression model. (**b**) Plot of the best gene selected by SVM-RFE algorithm. (**c**) Venn diagram embodying the eight diagnostic biomarkers common to both machine learning algorithms. LASSO, least absolute shrinkage and selection operator; SVM-RFE, support vector machine-recursive feature elimination.

The expression matrices of 23 AR-DEGs were used to construct the best diagnostic model using both least absolute shrinkage selection operator (LASSO) regression and support vector machine recursive feature elimination (SVM-RFE) algorithms to finally obtain potential diagnostic biomarkers of DCM. The LASSO regression algorithm narrowed down the range of AR-DEGs of DCM and obtained 9 variables as potential diagnostic biomarkers for DCM (Fig. 6a). The SVM-RFE algorithm was implemented to identify 13 signature genes (Fig. 6b).

Finally, 8 overlapping genes (*PLEKHF1*, *HSPG2*, *HSF1*, *TRIM65*, *DICER1*, *VDAC1*, *BAD*, *TFEB*) were obtained (Fig. 6c).

### Construction of transcription factor (TF)-gene regulatory network

**Figure 7:**
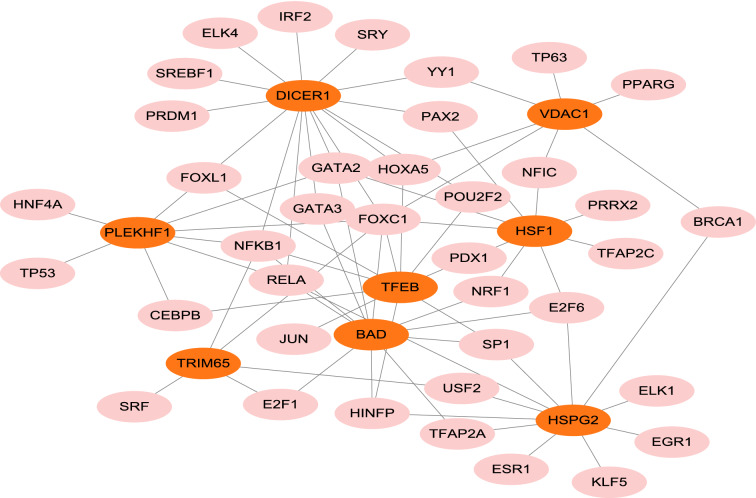
Network of transcription factors interacting with 8 potential diagnostic biomarkers. The highlighted orange nodes indicated the 8 potential diagnostic biomarkers and the other pink nodes indicated transcription factors. The network consisted of 8 core genes, 46 nodes and 76 edges.

Based on the JASPAR TF binding site profile database, TF-gene regulatory network was constructed using the NetworkAnalyst 3.0 platform. The TF-gene regulatory network was constructed based on 8 diagnostic biomarkers of DCM (*PLEKHF1*, *HSPG2*, *HSF1*, *TRIM65*, *DICER1*, *VDAC1*, *BAD*, *TFEB*) (Figure). The network included 46 loci with 76 edges. In detail, these loci are combined by 8 seed genes and 38 transcription factors. *TFEB* was regulated by 19 transcription factors and *DICER1* was regulated by 15 transcription factors. Figure 7 showed the TF-gene regulatory network.

### Gene targeted drugs screening

Based on the DSigNET drug database, the Enrichr (https://maayanlab.cloud/Enrichr/) web platform was used to identify drug molecules associated with 8 diagnostic biomarkers for DCM. Gene-targeted drugs were collected based on P-values. The combined score is proportional to the gene-drug association when the p-value is satisfied. The analysis showed that Melatonin CTD 00006260 and metformin CTD 00006282 had high gene binding to DCM. Table 3 listed the top 10 drugs for DCM by the DSigDB database.

**Table 3:** Drugs of choice for dilated cardiomyopathy.

Term	p-value	Combined score	Genes
Arsenenous acid CTD 00000922	5.77E−05	76.35849	*PLEKHF1*;*BAD*;*ADIPOQ*;*HSF1*;*TFEB*;*EIF4EBP1*;*CXCR4*;*PPFIA4*
Melatonin CTD 00006260	0.000058	462.545	*BAD*;*ADIPOQ*;*EIF4EBP1*
Metformin CTD 00006282	0.000115	338.4126	*BAD*;*ADIPOQ*;*EIF4EBP1*
Tretinoin HL60 UP	0.000152	154.1536	*RCAN1*;*GABARAPL1*;*CXCR4*;*PPFIA4*
Imatinib CTD 00003267	0.000231	244.7684	*BAD*;*HSF1*;*EIF4EBP1*
Arsenenous acid CTD 00000922	0.000374	61.03121	*GABARAPL1*;*HSF1*;*EIF4EBP1*;*CXCR4*;*VDAC1*;*TRIM65*
Wortmannin CTD 00000504	0.000606	154.125	*BAD*;*HSF1*;*EIF4EBP1*
Isoflupredone HL60 UP	0.000867	371.9284	*RCAN1*;*CXCR4*
Telmisartan CTD 00003021	0.001059	325.1773	*ADIPOQ*;*AGTR1*
Rosiglitazone CTD 00003139	0.001142	68.5849	*GABARAPL1*;*BAD*;*ADIPOQ*;*CXCR4*

### Genetic disease association analysis

**Figure 8:**
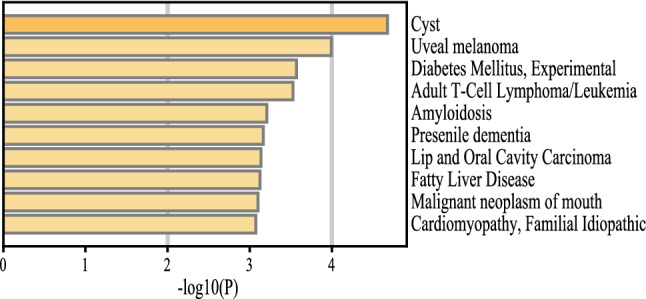
The process of identifying comorbidities in DCM.

Gene list enrichments were identified in the DisGeNET dataset. All genes in the genome had been used as the enrichment background. Terms with a p-value < 0.01, a minimum count of 3, and an enrichment factor > 1.5 (the enrichment factor is the ratio between the observed counts and the counts expected by chance) were collected and grouped into clusters based on their membership similarities. The top 10 enriched clusters were shown in the Fig. 8. The algorithm used here was the same for pathway and process enrichment analysis. Cyst, Uveal melanoma, Diabetes Mellitus, Experimental, Adult T-Cell Lymphoma/Leukemia, and Amyloidosis were identified as top 5 comorbidities of DCM.

## Discussion

It is well known that DCM is impaired ventricular dilation and systolic diastole, leading to arrhythmias and heart failure in severe cases. Unfortunately, with the low prevalence of EMB, most patients with early-stage cardiomyopathy are not effectively treated. The gold standard for myocarditis and DCM is often poor prognosis in cases of concomitant arrhythmias and heart failure^2^. Therefore, early diagnosis, precise evaluation, and therapeutic management of patients with DCM appear crucial. Hence, researchers are increasingly looking for diagnostic markers of DCM. Meanwhile, the molecular pathogenesis of DCM, viral infections, and other factors in disease progression and prognosis are still incompletely studied^15^.

It is well known that autophagy plays an important role in cancer, neurodegenerative diseases, inflammatory diseases, and cardiac diseases^5^. Among these, autophagy mechanisms are increasingly studied in cardiac diseases, and autophagy plays a crucial role in maintaining typical cardiac structure, function, and therapy^16,17^. Two key autophagy-related molecules, mTOR and Beclin1, had been shown to play a regulatory role in myocardial ischemia–reperfusion injury^17^. Among them, mTOR is involved in the *PI3K* and *Akt* pathway to regulate myocardial ischemia/reperfusion-induced apoptosis and autophagy^18^. In addition, Beclin1 exerts a positive impact on myocardial ischemia and an adverse effect during myocardial ischemia/reperfusion^19^. Currently, studies on the role of autophagy in cardiomyopathy-related diseases are increasing^13^, and research has shown that damage to the autophagic lysosomal pathway (ALP) and activation of inflammatory vesicles were important factors contributing to DCM^14^. Improved left ventricular size and cardiac function in mice with DCM deficient in *NCOA4* (nuclear receptor coactivator 4, an autophagy-associated gene that mediates ferritin degradation) inhibit free ferrous iron overload and increased lipid peroxidation^20^. Carolina et al.^21^ found that autophagy-related genes, such as *CALCOCO2* and *NRBP2*, the former of which regulates the expression of the latter, adversely affected left ventricular function parameters in patients with DCM.

In recent years, the exploration of the diagnostic and prognostic role of genetic biomarkers targeting DCM has been on the rise. For example, *CYR61* and *APN* were identified as two target genes for DCM by gene expression profiling studies in the GSE4172 dataset raw data^22^. It had been shown that *RBM20* induced aberrant TNN splicing as a determinant of DCM and increased the risk of arrhythmias^23^. In previous bioinformatics studies, genes or transcription factors such as *CTGF*, *POSTN*, *CORIN*, and *FIGF* were closely associated with DCM^24^. However, few studies have been conducted on the value of autophagy-related genes in diagnosing DCM.

To the best of our knowledge, this study is the first to investigate the diagnostic role of ARGs in DCM by mining the GEO database and integrating machine learning and bioinformatics approaches. We used the NetworkAnalyst 3.0 platform to deeply analyze the GSE4172 dataset, which compares gene expression in DCM with healthy samples infected by the fine virus B19. Using differential analysis, we obtained 770 DEGs and combined them with the gene set from the autophagy databases to obtain 23 AR-DEGs of DCM. Finally, by machine learning methods such as LASSO regression and SVM-RFE, we obtained 8 (*PLEKHF1*, *HSPG2*, *HSF1*, *TRIM65*, *DICER1*, *VDAC1*, *BAD*, *TFEB*) diagnostic biomarkers of DCM. Previous studies showed significant relevance regarding DCM or cardiomyocyte remodeling in the above eight genes.

*PLEKHF1* (Pleckstrin homology and FYVE domain containing 1) is located in the lysosome and plays a vital role in caspase-independent apoptosis, a process involved in autophagy^25^. In previous studies, *PLEKHF1* is a susceptibility gene for several diseases. For example, Qi et al., identified *PLEKHF1* as a potential biomarker for diabetic atherosclerosis^26^; also, *PLEKHF1* was shown to be a potential biomarker for chronic graft-versus-host disease, the accuracy of which was confirmed by several clinical independent validation studies^27^. In addition, it had been shown that levosimendan ameliorated myocardial infarction and ventricular remodeling in diabetic rats, and the expression of the gene *Plekhf1* received regulation by levosimendan, showing the potential of *Plekhf1* as a target gene for myocardial infarction and diabetic cardiomyopathy^28^.

*HSPG2* (Heparan sulfate proteoglycan 2) plays an important role in cancer growth, development, and metastasis^29^. Previous studies had shown that *HSPG2* was identified in key cardiac-related regions controlled by chromosome 1p36^30^, and related studies had demonstrated that chromosome 1p36 deletion was responsible for cardiovascular malformations and cardiomyopathy^31^, suggesting an important role for *HSPG2* in the pathogenesis and prognostic impact of cardiomyopathy^30^. In addition, *HSPG2* also plays an independent predictive role in a variety of diseases. For example, *HSPG2* was overexpressed in acute myeloid leukemia and can be used as a prognostic biomarker^32^. Recent studies had shown that *HSPG2* deficiency was a risk factor for aortic coarctation^33^.

*HSF1* (Heat shock transcription factor 1) is a significant heat stress response factor that plays an important role in inhibiting apoptosis and pathological remodeling of cardiomyocytes and is a protective factor for cardiomyocytes. In a previous quantitative transcriptomic analysis, *HSF1* was found to be significantly enriched in cardiomyocytes^34^. It had been shown that *HSF1* could be isolated by the death trap method, preventing hydrogen peroxide-induced cardiomyocyte death. It was found that overexpression of *HSG1* in transgenic mice reduced ischemia–reperfusion-induced cardiomyocyte injury^35^. In the present study, *HSF1* expression was lower in the DCM group compared with the healthy control group, which was also consistent with the findings of previous studies. In addition, it had been shown that overexpression of *HSF1* in *BAG* mutation-associated DCM helped to attenuate pathological remodeling of cardiomyocytes and alleviate proteostatic stress^36^. In contrast, recent studies had shown that *HSF1* overexpression lead to reduced expression of myofilament localization-associated *BAG3*. Decreased expression of *BAG3* was strongly associated with non-inherited heart failure and was more susceptible in male patients with DCM^37^. Therefore, the study of relevant molecules and pathways targeting *HSF1* contributes to our understanding of DCM.

*TRIM65* is an E3 ubiquitin ligase involved in the positive regulation of autophagy and was expressed in vascular endothelial cells, located in the cytoplasmic lysate and nucleoplasm. Unfortunately, there are relatively few studies related to *TRIM65*. From the available literature, it appeared that *TRIM65* was mainly involved in proteopathy and ubiquitination regulation to regulate disease progression and as a target for a variety of diseases^38,39^. Interestingly, although fewer studies are addressing the mechanisms associated with *TRIM65* and DCM, according to recent studies, *TRIM65* was closely linked to the inflammatory vesicle *NRLP3*^40^, which is known to play a role in a variety of DCM^14^. *TRIM65* was associated with antiviral innate immune mechanisms^41^. In addition, it had been shown that *TRIM65* regulated *VCAM-1* to control inflammatory responses^42^. All these studies point the way to exploring the molecular mechanism of *TRIM65* and DCM.

*DICER1* is a member of the ribonuclease III (RNaseIII) family and is involved in the production of microRNAs, which regulate gene expression at the post-transcriptional level and are more frequently studied in oncological diseases^43^. Evidence suggested that *DICER* deletion resulted in a dramatic decrease in the level of miRNAs it regulates, which led to severe DCM and heart failure in mice, a trend that was also seen in the expression of *DICER* proteins in diseased populations, implying an important role of *DICER* family genes in the pathogenesis of DCM^44^. Follow-up studies had shown that microRNAs act as negative regulators of genes and that specific regulation of microRNA expression could inhibit the loss of cardiac function due to *DICER* deficiency^45,46^, leading to cardioprotection. These studies suggested that endogenous microRNA competitive regulation of *DICER* family genes will be an essential strategy for gene targeting therapy in DCM.

*VDAC* (voltage dependent anion channel), including *VDAC1* and *VDAC2*, is a mitochondrial outer membrane pore-forming protein present in all eukaryotes. As a mitochondrial transporter protein, VDAG is mostly expressed in cardiac tissue and has significant tissue specificity^47,48^. It is well known that Ca^2+^ played a detrimental role in heart failure and myocardial ischemia/reperfusion, and Ca^2+^ overload activated the complex matrix chaperone procyclin D (CypD), which regulated the *VDAC1*, Grp75, and *IP3R1* complex and thus caused damage to cardiomyocytes, whereas inhibition of the CypD, *VDAC1*, Grp75, and *IP3R1* complex could protect cardiomyocytes^49^. Numerous studies had shown^50,51^ that regulation of *VDAC1* expression through microRNA targeting could regulate mitochondrial function and promoted the release of mitochondrial calcium for cell protection. Furthermore, in DCM mice, the lncRNA H19/miR-675 axis competitively downregulated *VDAC1*, reducing apoptosis. The above report provides a new strategy to explore the role of *VDAC1* in DCM. It was shown that *VDAC1* expression was upregulated in the hearts of patients with hypertrophic cardiomyopathy^52^. In the present study, the expression of *VDAC1* was also upregulated in samples from patients with DCM. These findings could explain the unique role played by *VDAC1* as a target gene for DCM.

*BAD* (Bcl-2 associated agonist of cell death) often follows Bcl-2 and plays an anti-apoptotic role. In a TNF-α-mediated mouse model of DCM in which apoptosis occurs, the expression of *BAD* was reduced in association with Bcl-2d^53^, which was consistent with the findings of the present study. According to previous studies, *BAD* played a key role in inducing β-cell apoptosis in Friedreich's ataxia, a neurodegenerative disease closely related to cardiomyopathy and diabetes^54^. It is well known that microRNAs regulate protein expression of mRNAs through negative regulation and play an important role in cardiovascular diseases, especially in heart failure and cardiac remodeling^55^. Studies had shown that multiple microRNAs played a regulatory role on *BCL2*^56^ and all of them were upregulated in heart failure^57^. As an antagonist of apoptosis, the protective role of *BAD* and Bcl-2 in the pathogenesis of DCM depended on further studies.

*TFEB* (transcription factor EB), a transcription factor located within the cytoplasmic lysosol (cytosol), is the master gene of the autophagic machinery of lysosomal biogenesis and coordinates the autophagic process, including autophagosome formation, autophagosome-lysosome fusion, and substrate degradation by driving the expression of autophagy and lysosomal genes^58^. According to reports, *TEFB* expression was highest in 18-week-old fetal heart tissue, with significant tissue specificity^59^. There is growing evidence that *TFEB* plays an important role in various types of DCM. Lysosomal storage disorders (LSD) lead to cardiac involvement in hypertrophic cardiomyopathy and DCM^60^. Further studies had shown that the Yes-associated protein (YAP) and Feb signaling pathway played a role in LSD disease by eliminating autophagic lysosomes, reducing cell death, and restoring cardiac function^61^. Also, it was found that *TFEB* deficiency led to cardiomyocyte hypertrophy and DCM causing heart failure^62^. Therefore, the role of *TFEB* in targeting DCM is extremely significant.

In addition, we performed a functional enrichment analysis of the pathogenesis of DCM and related molecular pathways and found that AR-DEGs of DCM were mainly enriched in autophagy regulatory pathways and cell growth signaling, such as regulation of autophagy, macroautophagy, AMPK signaling pathway, PKB-mediated events, etc. AMPK (Adenosine monophosphate-activated protein kinase) signaling pathway had been reported to be an important intracellular signaling pathway in the heart^63^. As an emerging target recognized for the treatment of heart failure^64^, AMPK plays an important role in regulating cardiomyocyte growth^65^. Numerous studies had shown that the AMPK pathway and its binding autophagy-related pathways played a protective role in the pathological development of cardiomyopathy^66–69^. These studies have provided ideas to explore the mechanistic studies of autophagy-related DCM. *PKB* (protein kinase B), also known as serine/threonine kinase Akt, serves as a central node for a variety of biological processes^70^. It had been reported that *PKB* was involved in protective mechanisms against myocardial ischemia/reperfusion^71^. However, relatively few studies have been conducted on the association of PKB-mediated events with DCM. According to previous studies, Pleiotrophin, a pro-angiogenic factor, was significantly expressed in rat models of myocardial infarction and DCM patients. It is considered that Pleiotrophin protects the myocardium by inhibiting endogenous AKT/PKB activity^72^. In contrast, Alexander et al. found that PKB phosphorylation expression restored cardiac contractility in a zebrafish model of DCM^73^.

In addition, we constructed TF-gene regulatory networks based on 8 autophagy-related genes in DCM and predicted them to target drugs, such as Melatonin and metformin. Studies showed that Melatonin had a better inhibitory effect on left heart dysfunction and ventricular remodeling in DCM rats with cardiorenal syndrome^74^. Metformin was able to partially reverse ventricular remodeling in mice with DCM through an autophagic mechanism^75^. These studies provided a basis and direction for clinical precision targeting therapy and novel drug development in DCM. In addition, we explored the comorbidities associated with DCM, such as fatty liver disease. Some scholars found that^76^ NAFLD affected the cardiovascular system through metabolic and inflammatory responses, and also increased the abnormalities of cardiac anatomy including cardiomyopathy^77^. Furthermore, the disease pathways between the two need further investigation.

However, there are certain shortcomings in our study. First, our data set of DCM was mined and analyzed secondarily by bioinformatics means, and the results of the study need to be validated with external evidence. In addition, the results of this study need to be combined with single-cell sequencing as the multi-omics study progresses. Finally, the mechanism of action and interrelationship between these 8 DCM genes and autophagy-related genes need further investigation.

## Methods

### Dilated cardiomyopathy dataset acquisition

The dataset of DCM was downloaded from the GSE4172 dataset of the Gene Expression Omnibus (GEO) (https://www.ncbi.nlm.nih.gov/geo) database, which was contributed by Wittchen et al.^22^, piggybacked on the GPL570 [HG- U133_Plus_2] platform using Affymetrix Human Genome U133 Plus 2.0 Array, containing eight endomyocardial myocardial biopsy samples from patients with microvirus B19-associated cardiac inflammation as experimental group and four healthy human samples as a control group. Clinical information of patients from the GSE4172 dataset was presented in Table 4.

**Table 4:** Clinical information for the GSE4172 dataset.

Sample	Group	Age	Gender	Ejection fraction	Left ventricular end diastolic diameter	Inflammation/PVB19
GSM94836	DCM	45	Male	34	62	Positive
GSM94837	DCM	62	Male	51	73	Positive
GSM94838	DCM	31	Male	52	57	Positive
GSM94839	DCM	67	Male	43	59	Positive
GSM94840	DCM	60	Male	34	76	Positive
GSM94841	DCM	69	Male	35	60	Positive
GSM94842	DCM	55	Female	31	61	Positive
GSM94843	DCM	31	Female	56	71	Positive
GSM94831	Healthy control	36	Female	68	47	Negative
GSM94854	Healthy control	46	Female	61	49	Negative
GSM94855	Healthy control	26	Female	74	47	Negative
GSM94870	Healthy control	36	Male	64	50	Negative

### Autophagy genes acquisition

A total of 232 autophagy genes were downloaded from the Human Autophagy Database (HADb, http://autophagy.lu/). Similarly, 796 autophagy genes were obtained from the Human Autophagy Modulator Database (HAMdb, http://hamdb.scbdd.com)^78^. A total of 803 autophagy-related genes were obtained as the autophagy gene set for this study by taking the intersection of the two.

### Identification of differentially expressed genes (DEGs) in autophagy-related genes (ARGs)

NetworkAnalyst 3.0 is a user-friendly bioinformatics visualization web platform for transcriptome analysis, gene network construction, and meta-analysis of gene expression data^79^. The expression data and grouping information of the GSE4172 dataset were submitted to NetworkAnalyst 3.0 for identification of the DCM groups and the healthy control groups for DEGs. For mRNA in microarrays, the threshold was set to |log2FoldChange|≥ 0.8 with a p value < 0.05, and genes meeting this criterion were considered as DEGs. We used the ggplot2 package (R package version 4.1.3) and pheatmap package (R package version 4.1.3) to draw the asymptotic volcano map and heatmap to show the DEGs. Autophagy-related genes (ARGs) and DEGs from the GSE4172 dataset were taken to intersect to obtain the set of autophagy-related differentially expression genes (AR-DEGs). Venn plots were created by using the Omicshare online tool (https://www.omicshare.com/). The expression of 23 AR-DEGs in GSE4172 was demonstrated using box plots through the ggpubr package as well as the associated helper R packages. The correlation analysis of AR-DEGs was visualized using the corrplot package (R package 4.1.3).

### Functional enrichment analysis

Functional enrichment consists of performing biological processes, molecular functions, and chromosomal location analysis^80^. Gene annotation uses gene ontology (GO) terminology and consists of biological processes, molecular functions, and cells. The Kyoto Encyclopedia of Genes and Genomes (KEGG) pathway was used to understand metabolic pathways and plays an important role in the gene annotation process^81,82^. In addition, the BioCarta, WikiPathways^83^, and Reactome^84^ databases were also used to analyze KEGG pathways. The Enrichr (https://amp.pharm.mssm.edu/Enrichr/) platform provides a comprehensive gene enrichment analysis applied databases containing rich gene set annotation, pathway information analysis, and screening of gene target drugs^85,86^. The GO terms of the AR-DEGs of DCM and all pathway information for this study were obtained from the Enrichr platform.

### Machine learning identifies molecular markers of AR-DEGs in DCM

In this study, the least absolute shrinkage and selection operator (LASSO) logistic regression was used for feature gene selection to reduce the number of genes in the disease prediction model, solve the multicollinearity problem in the regression analysis, and screen the molecular markers of DCM genes^87^. The "glmnet" package was used to implement the LASSO regression algorithm with α set to 1 which was used to control the traits of the model when dealing with highly correlated data. In addition, the Support Vector Machine-Recursive Feature Elimination (SVM-RFE) algorithm model was also used in this study to characterize the AR-DEGs and remove irrelevant genes to make the diagnostic prediction model more robust^88^. The SVM-RFE was implemented by the e1071 Package R software.

### Transcription Factor (TF)-gene regulatory network construction

The JASPAR (http://jaspar.genereg.net/) database was used to generate a visual analysis of the TF-gene co-regulatory network^89^. Based on 8 biomarkers of DCM, TFs that regulated the activity of functional pathways and gene expression levels in DCM were identified from the JASPAR database to form the TF-gene regulatory network. It is important to note that the JASPAR database is included in the NetworkAnalyst 3.0 platform.

### Target drug screening

Gene target-based drug screening has become a new approach for drug molecular identification study, which helps to expand the scope of relevant drugs and reduce the process of drug development. In this study, molecular markers of DCM were screened for drug candidates through the drug Signatures database (DSigDB), which consists of 17,389 drugs and 19,531 genes associated with the drugs^90^. The DSigDB database can be accessed by visiting Enrichr (https://www.amp.pharm.mssm.edu/Enrichr/) website to enter relevant gene targets and download target drug information. Drugs with p-values less than 0.05 and with larger combined scores were considered to be typically significant. The combined score represents the degree to which the small molecule drug is closely linked to the gene of interest.

### Genetic disease association analysis

The DisGeNET (http://www.disgenet.org) database is an open and versatile platform for studying specific human diseases and their comorbidities through genetic and molecular pathways, probing the characteristics of disease genes and offering the possibility to elucidate the mechanisms of disease^91^. In the present study, molecular markers of DCM were uploaded to the Metascape (https://metascape.org/gp/index.html#/main/step1) platform^92^, which contains the DisGeNET database. We have revealed DCM-related comorbidities through the DisGeNET database, laying the foundation for the mechanistic study of DCM.

### Copyright permission of KEGG

We have contacted Kanehisa Laboratories. We do not directly use these KEGG Pathway map “images” in the article, we need not obtain copyright permission of KEGG. However, they believe that we have written our article using their data, they kindly ask us to cite the following articles in it^81,93,94^.

## Supplementary Information

Supplementary Information.

## Data Availability

The dataset GSE4172 for this study can be found in the GEO database (https://www.ncbi.nlm.nih.gov/geo). All data generated or analysed during this study are included in this published article.

## Abbreviations

DCM: Dilated cardiomyopathy
ARGs: Autophagy-related genes
GEO: Gene Expression Omnibus
DEGs: Differentially expressed genes
HADb: Human Autophagy Database
HAMdb: Human Autophagy Modulator Database
AR-DEGs: Autophagy-related differentially expressed genes
GO: Gene ontology
DSigDB: Drug Signatures database
KEGG: Kyoto Encyclopedia of Genes and Genomes
EMB: Endomyocardial biopsy
BP: Biological process
CC: Chromosomal location
MF: Molecular function
LASSO: Least absolute shrinkage and selection operator
SVM-RFE: Support vector machine-recursive feature elimination
TF: Transcription factor
ALP: Autophagic lysosomal pathway
CypD: Chaperone procyclin D
cytosol: Cytoplasmic lysosol
LSD: Lysosomal storage disorders
YAP: Lysosomal storage disorders
