# Supplementary material for: Machine learning and bioinformatics to identify 8 autophagy-related biomarkers and construct gene regulatory networks in dilated cardiomyopathy
Source: Sci Rep. 2022 Sep 2;12:15030. doi: 10.1038/s41598-022-19027-5 (PMC9440113; doi:10.1038/s41598-022-19027-5)
Supplement: Supplementary file 1 — Supplementary Information. [file 41598_2022_19027_MOESM1_ESM.docx]

**Machine learning and bioinformatics to identify 8 autophagy-related biomarkers and construct gene regulatory networks in dilated cardiomyopathy**

Fengjun Zhang^1†^, Mingyue Xia^2†^, Jiarong Jiang^3^, Shuai Wang^4^, Qiong Zhao^5^, Cheng Yu^6^, Jinzhen Yu^7^, Dexian Xian^1^, Xiao Li^8^, Lin Zhang^9^, Yuan Liu^5&*^, Min Peng^5&*^

Department of Orthopedics Trauma and Hand Surgery, The First Affiliated Hospital of Guangxi Medical University, Nanning, 530021, China

^1^College of Acupuncture and Massage, Shandong University of Traditional Chinese Medicine, Jinan, China.

^2^College of Traditional Chinese Medicine, Shandong University of Traditional Chinese Medicine, Jinan, China.

^3^Department of Cardiology, PLA Rocket Force Characteristic Medical Center, Beijing, China.

^4^Department of Pediatric Surgery, Shandong Provincial Hospital affiliated to Shandong First Medical University, Jinan, China.

^5^Department of Traditional Chinese Medicine, Shandong Provincial Hospital affiliated to Shandong First Medical University, Jinan, China.

^6^Department of Traditional Chinese Medicine, Shandong University of Traditional Chinese Medicine Affiliated Hospital, Jinan, Shandong, China.

^7^First Clinical Medical College, Shandong University of Traditional Chinese Medicine, Jinan, China.

^8^Department of Cardiology, Shandong University of Traditional Chinese Medicine Affiliated Hospital, Jinan, Shandong, China.

^9^Department of clinical pharmacy, Shaoxing People's Hospital, Shaoxing Hospital, Zhejiang University School of Medicine, Shaoxing, China.

^†^These authors contributed equally to this work.

^&^These authors also contributed equally to this work.

*Corresponding Author:

E-mail: pengmin186@126.com (MP); liuyuanly0429@163.com (YL).

**Supplement File 1** Related functions of 23 autophagy-related differentially expressed genes.

| Gene | Full name | Gene Function |
| --- | --- | --- |
| *ADIPOQ* | Adiponectin, C1Q And Collagen Domain Containing | *ADIPOQ* is expressed only in adipose tissue and encodes a protein similar to collagen X and VIII and complement factor C1q, which circulates in the plasma and is involved in metabolic and hormonal processes. *ADIPOQ* is involved in the control of lipid metabolism and insulin sensitivity and has direct anti-diabetic, anti-atherosclerotic and anti-inflammatory activities. |
| *TRIM17* | Tripartite Motif Containing 17 | The protein encoded by *TRIM17* is a mecmber of the tripartite motif (TRIM) family. The TRIM motif includes three zinc-binding domains, a RING, a B-box type 1 and a B-box type 2, and a coiled-coil region. The protein localizes to cytoplasmic bodies. The protein is expressed almost exclusively in the testis, but its function is unknown. |
| *PPFIA4* | PTPRF Interacting Protein Alpha 4 | *PPFIA4* belongs to the liprin-alpha gene family. *PPFIA4* regulates the disassembly of focal adhesions. |
| *CAPN12* | Calpain 12 | *CAPN12* belongs to the calpain large subunit family. Calpains are a family of cytosolic calcium-activated cysteine proteases involved in a variety of cellular processes including apoptosis, cell division, modulation of integrin-cytoskeletal interactions, and synaptic plasticity. |
| *PLEKHF1* | Pleckstrin Homology And FYVE Domain Containing 1 | *PLEKHF1* induces apoptosis through the lysosomal-mitochondrial pathway. *PLEKHF1* translocates to the lysosome and initiates lysosomal membrane (LMP) permeabilization, leading to the release of CTSD and CTSL into the cytoplasm. PLEKHF1 triggers cysteinase-independent apoptosis by altering mitochondrial membrane permeability (MMP), which leads to the release of PDCD8. |
| *RCAN1* | Regulator Of Calcineurin 1 | *RCAN1* is predominantly expressed in cardiac and skeletal muscle. the protein encoded by RCAN1 interacts with calcium-regulated neurophosphatase A and inhibits calcium-regulated neurophosphatase-dependent signaling pathways, potentially affecting central nervous system development |
| *RAB12* | RAB12, Member RAS Oncogene Family | *RAB12* is involved in several biological processes, including cellular response to insulin stimulation, endosomal to lysosomal transport, and cellular secretion. RAB12 regulates intracellular amino acid concentrations, mTOR complex activity, and autophagy. |
| *CXCR4* | C-X-C Motif Chemokine Receptor 4 | *CXCR4* encodes a CXC chemokine receptor specific for stromal cell-derived factor-1, which transduces signals by increasing intracellular calcium levels and enhancing MAPK1/MAPK3 activity. *CXCR4* leads to enhanced intracellular calcium and reduced cellular cAMP levels and is involved in hematopoiesis and cardiac septal formation. |
| *HSPG2* | Heparan Sulfate Proteoglycan 2 | *HSPG2* encodes perlecan protein, a key component of the extracellular matrix of the vasculature that helps maintain endothelial barrier function. It is a potent inhibitor of smooth muscle cell proliferation and is therefore thought to contribute to the maintenance of vascular homeostasis. |
| *EIF4EBP1* | Eukaryotic Translation Initiation Factor 4E Binding Protein 1 | The protein produced by *EIF4EBP1* translation interacts directly with eukaryotic translation initiation factor 4E (eIF4E), a restriction component of the multisubunit complex that recruits the 40S ribosomal subunit to the 5' end of mRNA. The interaction of *EIF4EBP1* with eIF4E inhibits complex assembly and suppresses translation. *EIF4EBP1* is phosphorylated in response to various signals, including UV irradiation and insulin signaling, leading to its dissociation from eIF4E and activation of mRNA translation |
| *HSF1* | Heat Shock Transcription Factor 1 | The product of *HSF1* is a transcription factor that rapidly induces and binds the heat shock promoter element (HSE) following temperature stress. This protein plays a role in regulating lifespan. the expression of *HSF1* is inhibited by phosphorylation, which promotes the binding of heat shock protein 90. |
| *ZC3H12A* | Zinc Finger CCCH-Type Containing 12A | *ZC3H12A* is an MCP1-inducible protein that acts as a transcriptional activator and causes cardiomyocyte death, possibly through the induction of genes associated with apoptosis. In the early stages of inflammation, *ZC3H12A* regulates the inflammatory response by promoting the degradation of a set of translationally active cytokine-induced inflammation-associated mRNAs, such as IL6 and IL12B. |
| *PRKAB1* | Protein Kinase AMP-Activated Non-Catalytic Subunit Beta 1 | The protein encoded by *PRKAB1* is a regulatory subunit of AMP-activated protein kinase (AMPK) and is a positive regulator of AMPK activity. myristoylation and phosphorylation of *PRKAB1* have been shown to affect the enzymatic activity and cellular localization of AMPK and also serves as an articulatory molecule mediating AMPK complex conjugation. |
| *TRIM65* | Tripartite Motif Containing 65 | *TRIM65* is located in the cytoplasmic lysate and nucleoplasm to enable zinc binding activity and participate in the positive regulation of autophagy. |
| *ARSA* | Arylsulfatase A | The protein encoded by *ARSA* hydrolyzes cerebroside sulfate to cerebroside and sulfate. defective *ARSA* causes heterochromatic leukodystrophy (MLD). |
| *GABARAPL1* | GABA Type A Receptor Associated Protein Like 1 | *GABARAPL1* promotes cis-intracellular transport of receptors to increase cell surface expression of kappa-type opioid receptors and participates in autophagosome vesicle formation. the GABARAP/GATE-16 subfamily is essential for the late stages of autophagosome maturation. |
| *DICER1* | Dicer 1, Ribonuclease III | The protein encoded by *DICER1* acts as a ribonuclease and is required for RNA interference and for the production of active small RNA components of the small time RNA (stRNA) pathway that inhibit gene expression. This protein also acts as a potent antiviral agent with activity against RNA viruses including Zika and SARS-CoV-2 viruses. |
| *VDAC1* | Voltage Dependent Anion Channel 1 | *VDAC1* encodes a voltage-dependent anion channel protein, which is a major component of the outer mitochondrial membrane. The encoded protein facilitates the exchange of metabolites and ions across the outer mitochondrial membrane and may regulate mitochondrial function. The protein also forms channels in the plasma membrane and may be involved in transmembrane electron transfer. |
| *CHMP4B* | Charged Multivesicular Body Protein 4B | *CHMP4B* is part of the endosomal sorting complex required for transport (ESCRT) complex III (ESCRT-III), which functions to sort endocytosed cell surface receptors into multivesicular endosomes. Mutations in this gene result in autosomal dominant posterior polar cataracts. |
| *AGTR1* | Angiotensin II Receptor Type 1 | *AGTR1* (angiotensin II receptor type 1) plays a role in generating reperfusion arrhythmias after restoring blood flow to ischemic or infarcted myocardium, and is an important target for the control of angiotensin II-dependent hypertension in the view of being involved in the pathogenesis of dilated or ischemic cardiomyopathy. |
| *BAD* | BCL2 Associated Agonist Of Cell Death | *BAD* promotes cell death and successfully competes for binding to Bcl-X(L), Bcl-2, and Bcl-W, thereby affecting the level of heterodimerization of these proteins with BAX. *BAD* reverses the death-inhibitory activity of Bcl-X(L) but not Bcl-2 (by similarity) and acts as a link between growth factor receptor signaling and the apoptotic pathway. |
| *TFEB* | Transcription Factor EB | *TFEB* (transcription factor EB) is a protein-coding gene. *TFEB* acts as a positive regulator of autophagy and promotes the expression of genes involved in autophagy and plays a role in signal transduction required for normal vascularization of the placenta. |
| *AP2M1* | Adaptor Related Protein Complex 2 Subunit Mu 1 | *AP2M1* encodes a subunit of the heterotetrameric shell assembly protein complex 2 (AP2), which belongs to the family of intermediate subunits of the junctional complex. The protein encoded by *AP2M1* may also play an important role in regulating intracellular transport and the function of CTLA-4 protein. |
